# Correction to ‘Wnt5b integrates Fak1a to mediate gastrulation cell movements via Rac1 and Cdc42’

**DOI:** 10.1098/rsob.200058

**Published:** 2020-03-18

**Authors:** I-Chen Hung, Tsung-Ming Chen, Jing-Ping Lin, Yu-Ling Tai, Tang-Long Shen, Shyh-Jye Lee

*Open Biol.* **10**, 190273. (Published Online 26 February 2020) (doi:10.1098/rsob.190273)

This correction refers to an error in the affiliation of author ‘Tsung-Ming Chen’. It should be:

^3^Department and Graduate Institute of Aquaculture, National Kaohsiung University of Science and Technology, Kaohsiung, Taiwan.

This has now been corrected.

