# Supplementary material for: Wnt5b integrates Fak1a to mediate gastrulation cell movements via Rac1 and Cdc42
Source: Open Biol. 2020 Feb 26;10(2):190273. doi: 10.1098/rsob.190273 (PMC7058935; doi:10.1098/rsob.190273)
Supplement: Supplementary Tables and Figures [file rsob190273supp1.pdf]

**SUPPLEMENTARY DATA**

Contains 3 tables and 22 figures.

**SUPPLEMENTARY Tables**

**Table S1.** Primers and MOs used in this study

| Name | Sequence from 5′ to 3′ | Description |
|---|---|---|
| *fak1a* F | AC*GGATCC*ATGGCAGCGGCTTACCTGG | For amplification of full |
| *fak1a* R | GT*GGATCC*TCACTGAGCTTTCTCCTCTGC | length Fak1a CDS |
| *fak1a* real time F | GGCTTCAAATCCACTACAG | |
| *fak1a* real time R | ATAATCCGCAGTGTTTAGC | |
| *frnk1a* | GGATCCAGCCGTATGGAGATGAGGA | For amplification of Frnk1a with Fak1a R |
| *fak1a* probe-1 | TGGTGAAGGAAGTGGGATTAG | For WISH probe synthesis |
| *fak1a* probe-2 | GCACAAGGCCCAAATATACAA | For WISH probe synthesis |
| *fak1a* $tMO_1$ | GTGGGTGCTAACTGTCCGTCATATT | For Fak1a knockdown |
| *fak1a* $tMO_2$ | TGATATCCTTGAAATATCGTATTCT | For Fak1a knockdown |
| *Fak1a* E10 F | AGGCTGAGAAAAGTACAAAA | For fak1a mutation screen |
| *Fak1a* E10 R | GAGTTGAGTGTAGTTTTGAAC | |
| *fak1b* $tMO_1$ | TGCCGTCGCCATGCCTTTACCGTAC | For Fak1b knockdown |
| *fak1b* $tMO_2$ | CTGGAAATATTGGTTTATCGAGTGA | For Fak1b knockdown |
| *Fak1b* F | ATATGGCGACGGCATTCCTGGAGC | For amplify full length fak1b and WISH probes |
| *fak1b* R | TCAGTGTGTGCGGTGGCTGTG | |
| *fak1b* real time F | GAAGACAAACCCACACTCAACTACT | For qPCR |
| *fak1b* real time R | CTGTAGAATCTCCTGATCTCCAGAC | |
| *Wnt5b* F | ATGGATGTGAGAATGAACCAA | For amplification of full length wnt5b CDS |
| *wnt5b* R | ATATGAGAGAAACACAGCTTGAG | |
| *wnt5b* real time F | AAACTCTTTAGAGACTCG | For qPCR |
| *wnt5b* real time R | GTGTGTTTAAGAATTGGAA | |
| *wnt5b* tMO | GTCCTTGGTTCATTCTCACATCCAT | For Wnt5b knockdown |
| *cdc42* real time F | GCTGATTGTTTGCCCACACT | |
| *cdc42* real time R | TCACACTCCTTTCCTCAGGC | For qPCR |
| *rac1* real time F | GCCCCTGCTCTTCAGTTCTG | |
| *rac1* real time R | GATCGTCTGCATAGCTGTGC | For qPCR |

**Table S2.** Guiding RNAs in this study

| Name | Sequence | Target exon |
|---|---|---|
| *fak1a* E3 -1 | GACCCATGCTCTGAACCAGG | Exon 3 |
| *fak1a* E3 -2 | GGCTAGCAACATCCGACACG | Exon 3 |
| *fak1a* E10 -1 | GGAGCTTGCCATCGGGCCTG | Exon 10 |

**Table S3.** Characterization of *fak1a* mutant alleles

| Allele Symbol | Affected nucleotides (nt) | Flanking sequence of indel | Effect on coding region | Protein Length in aa |
|---|---|---|---|---|
| Δ1 | 1 nt deletion | GTGGAGCTTGCCATCGGGC-TGAGGAGGGA | A frameshift at aa 275 with additional 7 aa afterward | 282 |
| Δ2a | 2 nt deletion | GTGGAGCTTGCCATCGGGC--AAGGAGGGA | A frameshift at aa 275 with additional 28 aa afterward | 303 |
| Δ2b | 2 nt deletion | GTGGAGCTTGCCATCGGGCCT--GGAGGGA | A frameshift at aa 276 with additional 27 aa afterward | 303 |
| Δ5a | 5 nt deletion | GTGGAGCTTGCCATC-----TGAGGAGGGA | A frameshift at aa 274 | 274 |
| Δ5b | 5 nt deletion | GTGGAGCTTGCCATCGGGC-----AAGGGA | A frameshift at aa 275 with additional 27 aa afterward | 302 |
| Δ6 | 6 nt deletion | GTGGAGCTTGCCATCGGG------GAGGGA | No frameshift | 1048 |
| Δ7 | 7 nt deletion | GTGGAGCTTGCCATCGGG-------AGGGA | A frameshift at aa 275 with additional 5 aa afterward | 279 |
| I1 | 1 nt insertion | GTGGAGCTTGCCATCGGGCAAGGAGGAGGGA | A frameshift at aa 275 with additional 29 aa afterward | 304 |
| l2 | 2 nt insertion | GTGGAGCTTGCCATCGGGCTGAG--GAGGAGGGA | A frameshift at aa 275 with additional 8 aa afterward | 281 |
| I7 | 7 nt insertion | GTGGAGCTTGCCATCGGGCTCTTATCCTGAGGAGGGA | A frameshift at aa 275 with additional 3 aa afterward | 277 |
| I16 | 16 nt insertion | GTGGAGCTTGCCATCGTGGTGATACACATCCCCTGGCCTGAGGAGGGA | A frameshift at aa 274 with additional 7 aa afterward | 281 |

*Δ: deletion; I: insertion; nt: nucleotide; -: deleted nucleotide; letter in red stands for inserted nucleotide; aa: amino acid

## SUPPLEMENTAL FIGURES & LEGEND

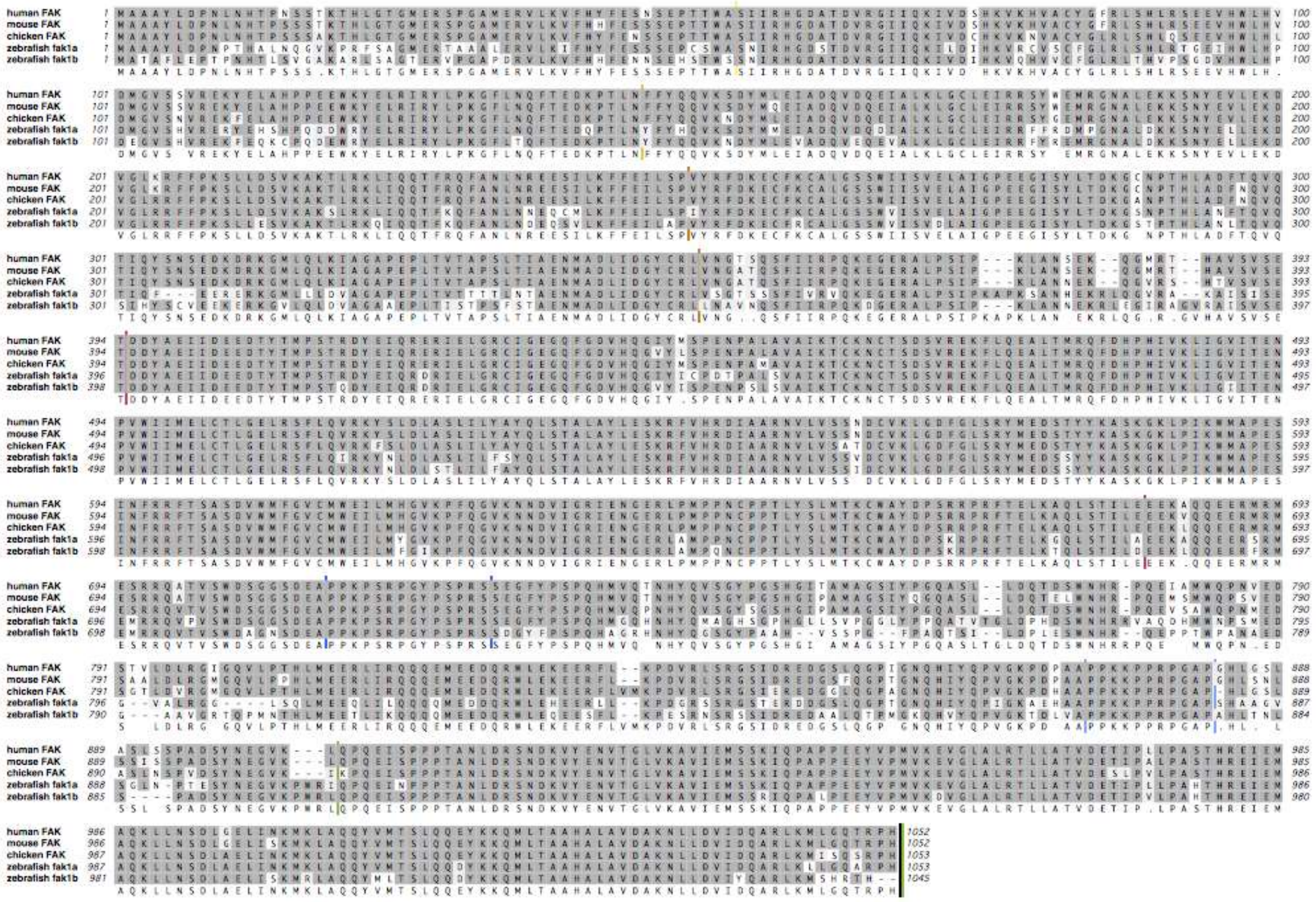


**Figure S1.** Amino acid sequence alignment of FAK proteins among different species. Amino acid sequences of zebrafish Fak1a (NP_571871.1) and Fak1b (NP_942114.1) were aligned with their orthologues of human (NP_775368.1), mouse (NP_766086.1) and chicken (NP_990766.1) using the Vector NTI software. Identical amino acids across all and some species are shaded in black and gray, respectively. Different color bars franking sequences corresponding to the functional domains depicted in Supplementary Figure S3.

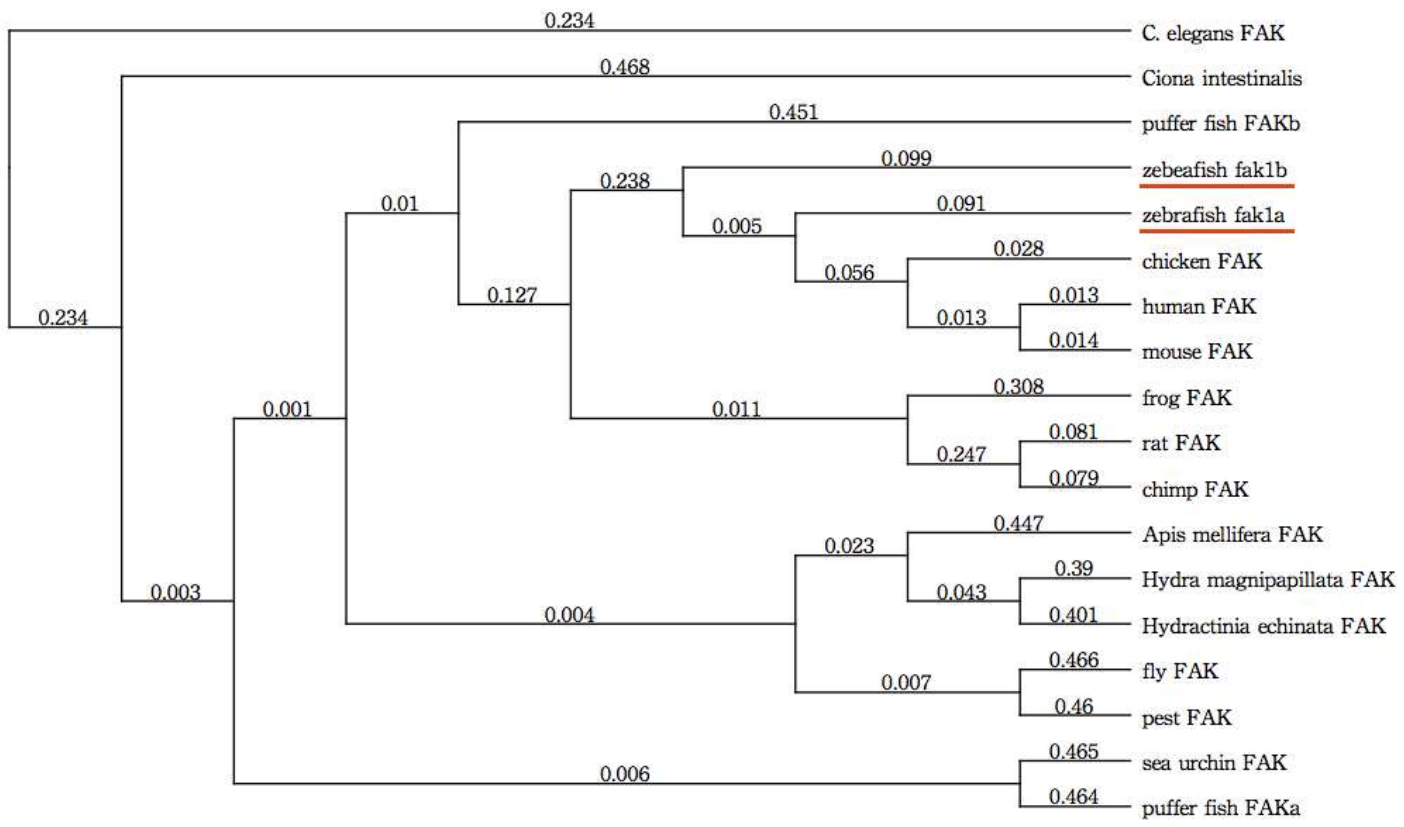


**Figure S2.** Phylogenetic analysis of FAK proteins among different species. **A** phylogenetic tree of FAK proteins was built based on amino acid sequences from different species according to the neighboring joint algorithm (MEGA 4.0). Bootstrap values were determined from 1,000 replicates and given for all present branches. The accession numbers of GenBank for various annotated FAK are: *C. elegans* (NP_740841.1), *Ciona intestinalis* (XP_002125758.2), puffer fish FAKb (XP_003968982.1), zebrafish Fak1a (NP_571871.1) and Fak1b (NP_942114.1), chicken (NP_990766.1), human (NP_775368.1), mouse (NP_766086.1), frog (NP_001084066.1), rat (NP_037213.1), chimpanzee (XP_519982.2), *Apis mellifera* (XP_001120873.2), *Hydra magnipapillata* (AAW21807.1), *Hydractinia echinata* (AAV97963.1), fly (NP_523793.2), pest (XP_309528.4), Sea urchin (AAN38839.1), puffer fish FAKa (XP_003966082.1).

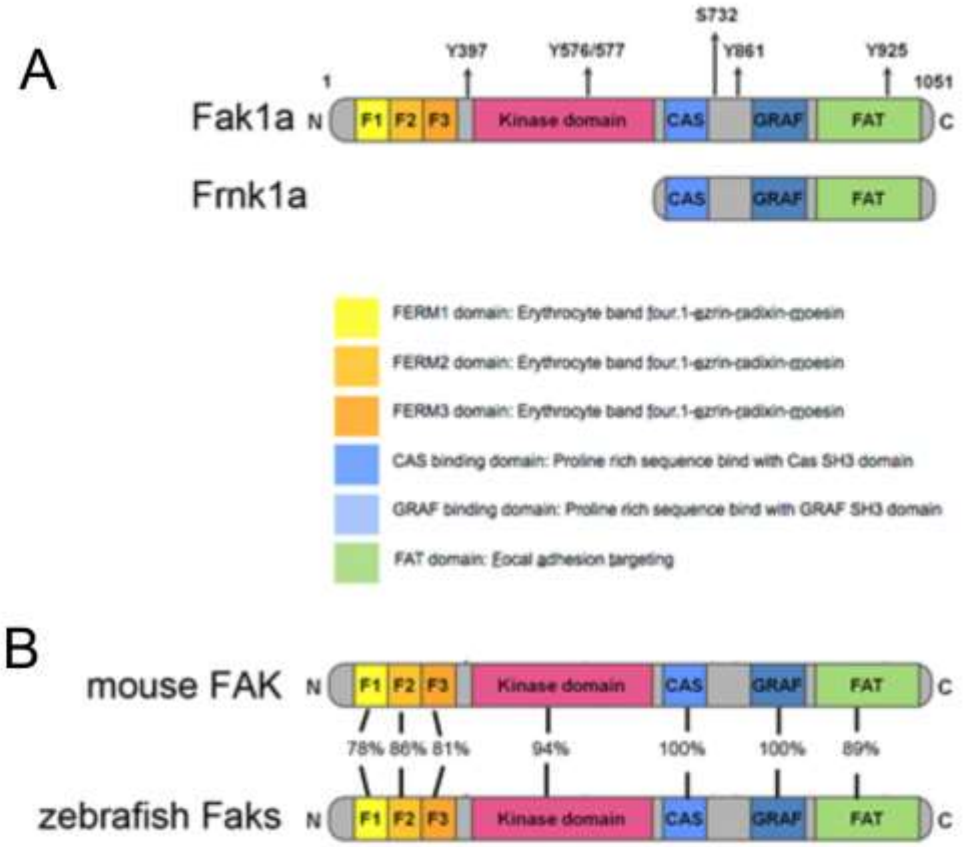


**Figure S3.** Domain and phosphorylation site characterization in FAKs. (A) Functional domains and conserved phosphorylation sites are shown in zebrafish Fak1a and Frnk1a. Different domains are painted with different colors and the description for each domain is shown below. The conserved phosphorylation sites are indicated by arrows. (B) The functional domains of zebrafish Faks (Fak1a and Fak1b) are compared to that of mouse FAK. The percentages of similarity between each domain are indicated.

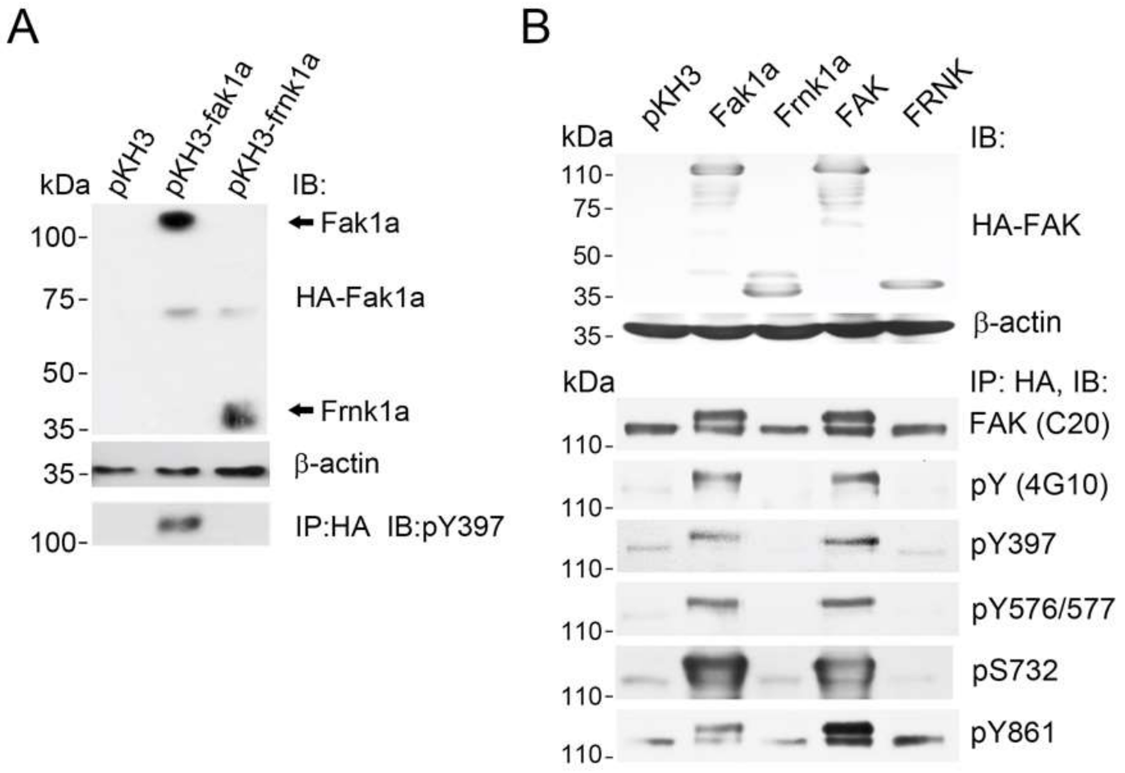


**Figure S4.** Zebrafish Fak1a has the conserved phosphorylation sites compared to that of chicken FAK. (A**)** Hemoagglutinin (HA)-tagged *fak1a* or *frnk1a* was transfected into FAK$^{-/-}$ MEF cells, immunoprecipitated (IP), and then subjected to immunoblotting (IB) against phospho-Tyr397. Both genes were successfully expressed as shown in the immunoblot using β-actin as an internal control. However, as shown in the HA-IP blot probing the FAK pY397 antibody, only Fak1a, but not Frnk1a, was autophosphorylated at Y397. (B) Zebrafish HA-tagged *fak1a*, *frnk1a*, chicken HA-tagged FAK, or FRNK was separately transfected and expressed in 293T cells, IP, and subjected to IB using antibodies against specific phosphorylation sites as designated. These data revealed that both zebrafish and chicken FAKs, but not FRNKs, could be autophosphorylated at Y397, Y576/577, S732, and Y861.

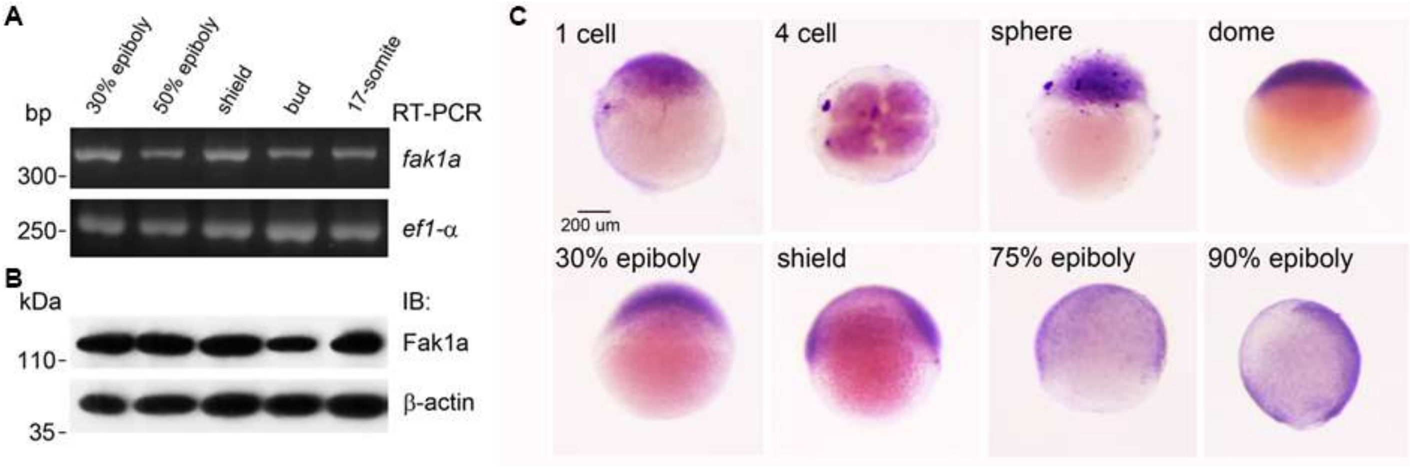


**Figure S5.** (A) RT-PCR (B) Western blotting (lower panel) against Fak1a in zebrafish embryos at indicated stage. *ef1α* and β-actin were used as internal controls for RT-PCR and Western blotting, respectively. (C) Whole-mount *in situ* hybridization against *fak1a* from 1-Cell to 90% epiboly stage embryos.

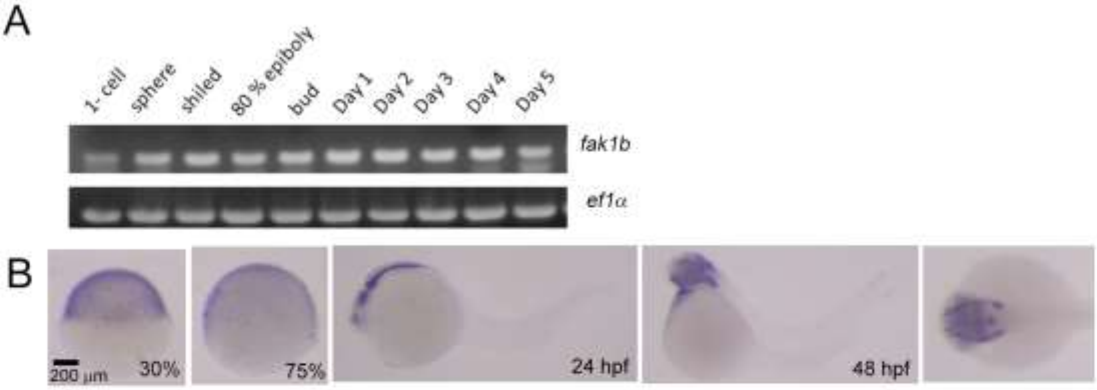


**Fig S6**. Temporal and spatial expression of *fak1b* at different stage. (A) RT-PCR against *fak1b* in zebrafish embryos at indicated stage. *ef1α* was used as an internal control. (B) Spatial expression pattern of *fak1b* by whole-mount *in situ* hybridization from 30% epiboly to 48 hpf. Lateral views are shown except an additional dorsal view of a 48-hpf embryo is shown on the right.

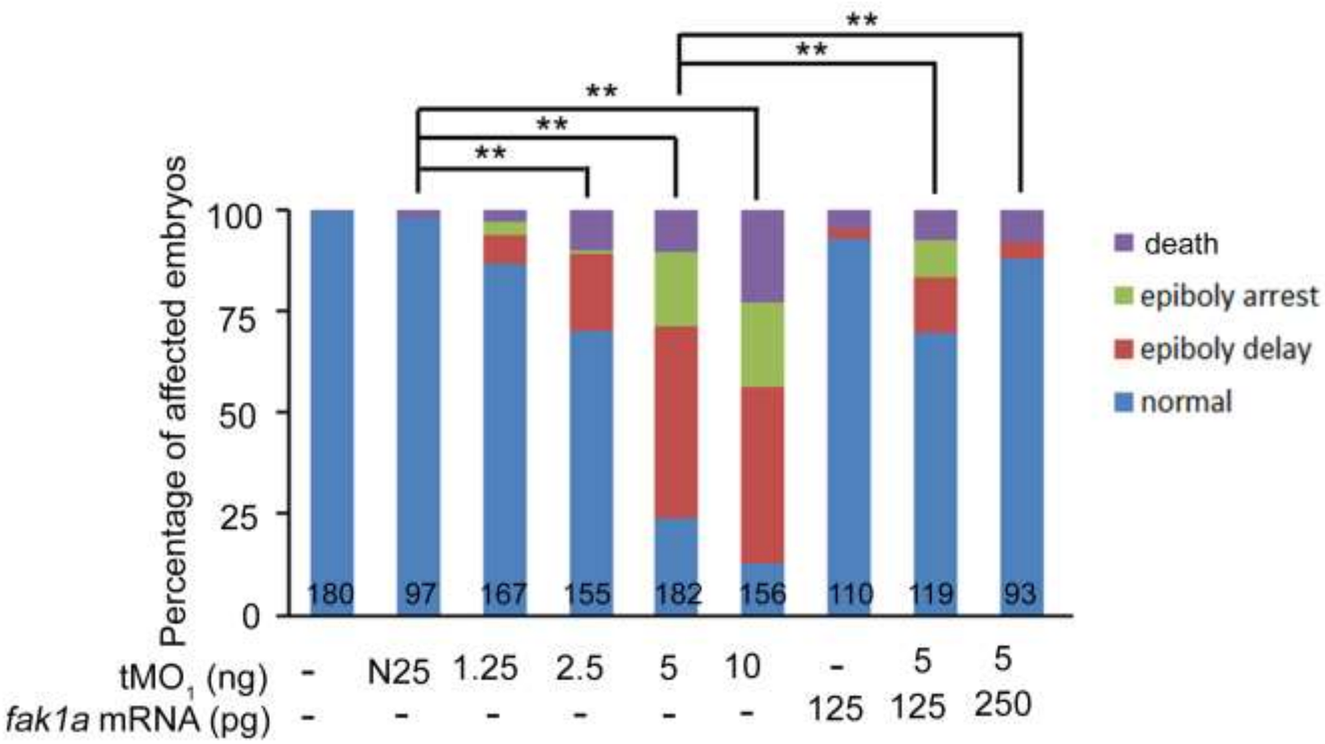


**Figure S7.** The *fak1a* translation blocking morpholino ($tMO_1$) causes dose-dependent and specific inhibition of epiboly. A dose-dependent disturbance of an epiboly defect was found in embryos injected with 1.25, 2.5, 5, and 10 ng per embryo of the *fak1a* $tMO_1$. The N25 MO at 10 ng was used as a control. Embryos were also co-injected with the *fak1a* $tMO_1$ and different amounts of *fak1a* mRNA and showed dose-dependent rescue of the epiboly defect ($n = 3$, ** $p < 0.01$, *** $p < 0.001$).

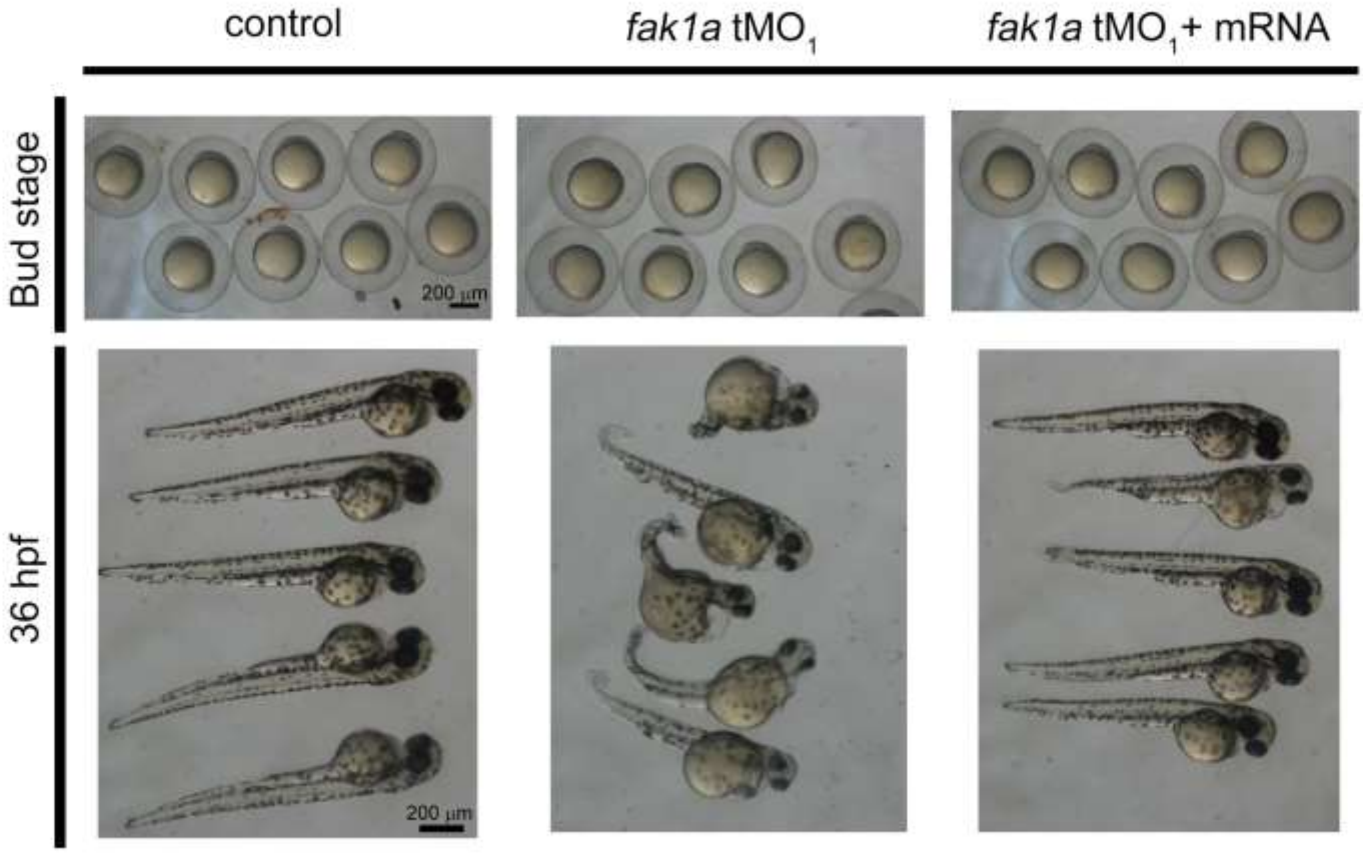


**Figure S8.** Overexpression of *fak1a* mRNA rescues gastrulation defects in *fak1a* morphants. Embryos injected with 5 ng of the *fak1a* translation blocking morpholino ($tMO_1$) with or without 200 pg of *fak1a* mRNA were cultured and photographed in the bud stage and at 36 hpf. The *fak1a* $tMO_1$-induced defects were notably rescued by *fak1a* mRNA.

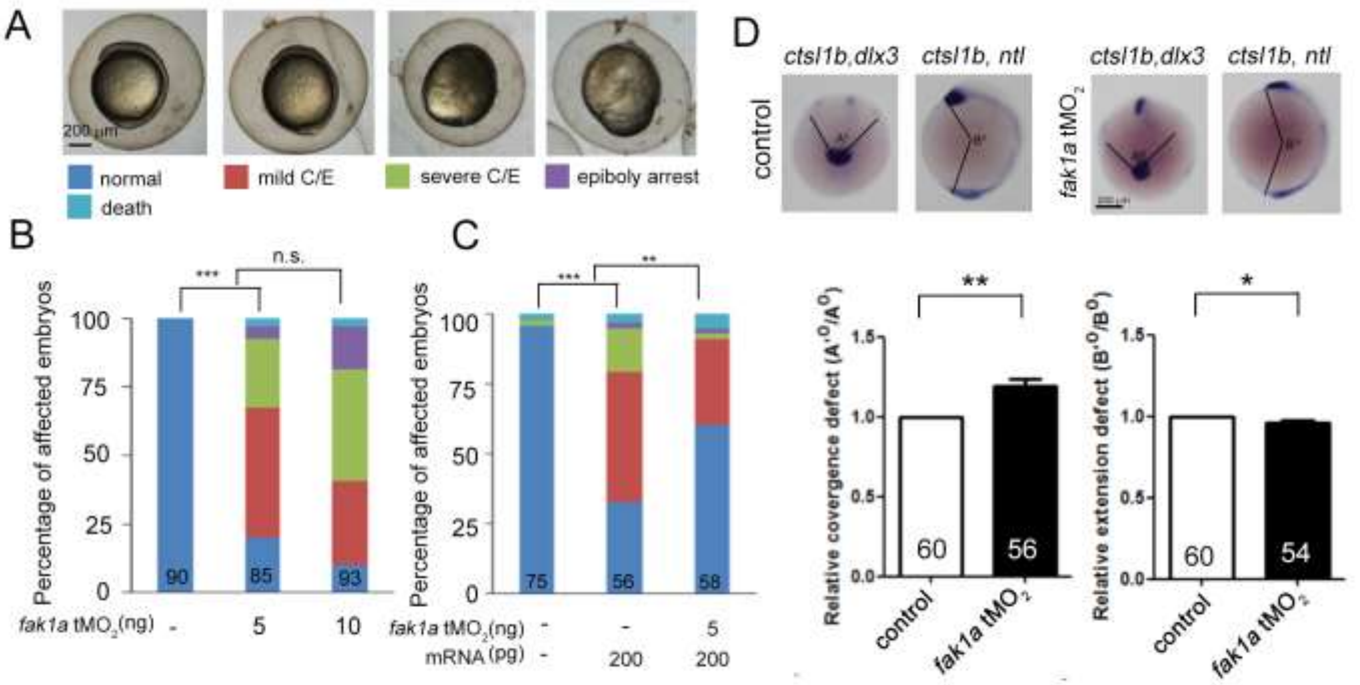


**Figure S9**. The *fak1a* translation blocking morpholino ($tMO_2$) causes dose-dependent and specific inhibition of gastrulation. (A) Embryos untreated or injected with 5 or 10 ng of the *fak1a* $tMO_2$ were cultured to the bud stage, and the resultant gastrulation defects were classified into normal, mild convergence and extension (C/E) defects, severe C/E defects, epiboly defects, and death. Representative photographs except for dead embryos are shown in side view with the dorsal to the right and the anterior to the top. Quantification data are shown in (B). (C) Embryos were untreated or injected with designated amount of the *fak1a* $tMO_2$ and *fak1a* mRNA, cultured, examined, and presented as previously described. (D) After examination, embryos were fixed and subjected to WISH against *ctsl1b/dlx3* or *ctsl1b/ntl* staining as shown in D. Quantitative data of relative convergence (A'°/A°) and extension (B'°/B°) defects between morphants and untreated control embryos are shown in the lower bar graphs. The number of embryos analyzed are presented at the bottom of each column. * $p < 0.05$, ** $p < 0.01$, $n = 3$.

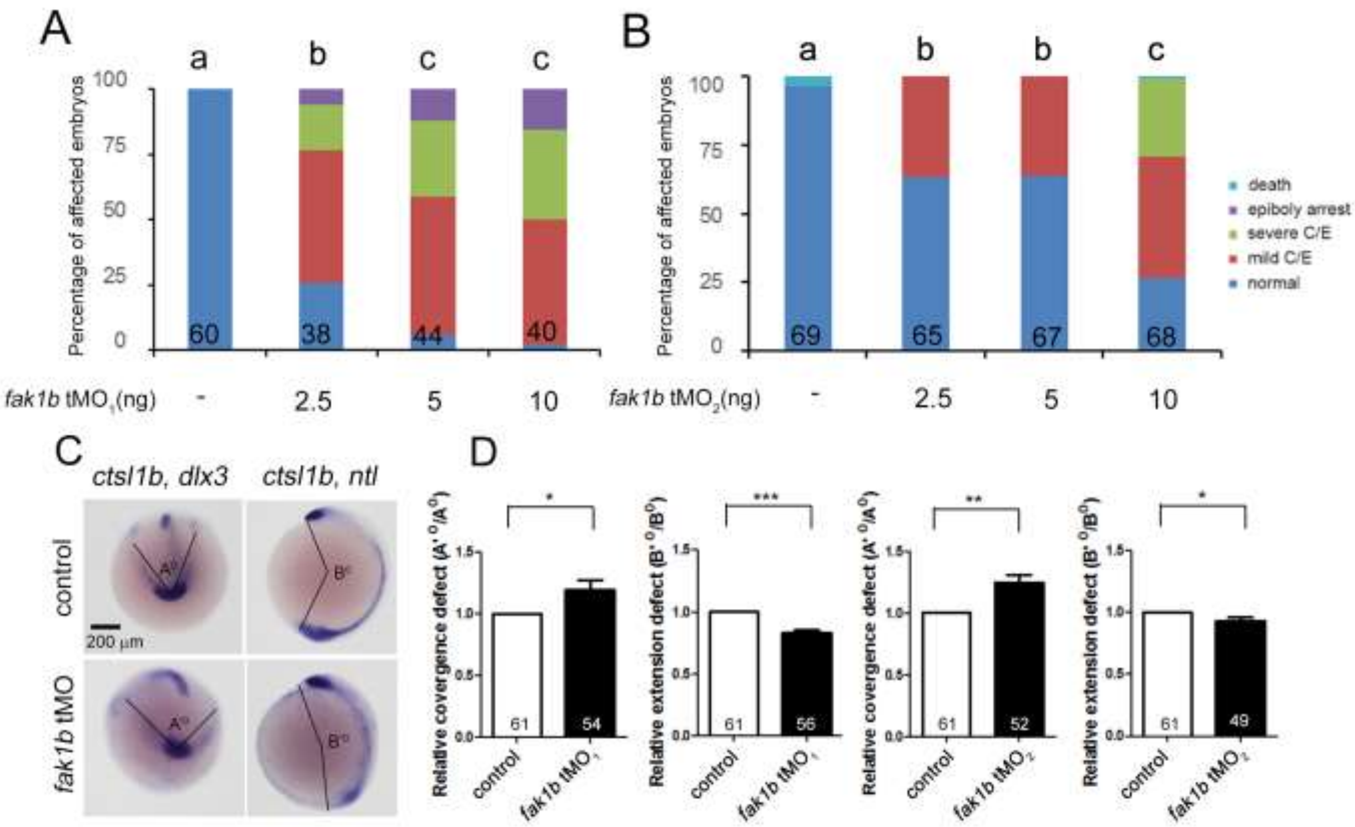


**Figure S10.** Loss of Fak1b causes dose-dependent inhibition on epiboly. Embryos injected without or with designated amount *fak1b* $tMO_1$ (A) or *fak1b* $tMO_2$ (B) were observed during gastrulation. Quantitative data of *fak1b* $tMO_1$ morphants are shown according to the classification mentioned previously. Values between groups with a significant difference ($p < 0.05$) are denoted by different letters. (C) Representative photographs of embryos injected without (control) and with 5 ng *fak1b* $tMO_1$ are shown. (D) Quantitative data of relative convergence ($A'^0/A^0$) and extension ($B'^0/B^0$) defect between *fak1b* $tMO_1$ (5 ng) or $tMO_2$ (10 ng) morphants and controls are shown. The number of embryos analysed are presented in the bottom of each column. *$p < 0.05$, **$p < 0.01$, ***$p < 0.001$, N = 3.

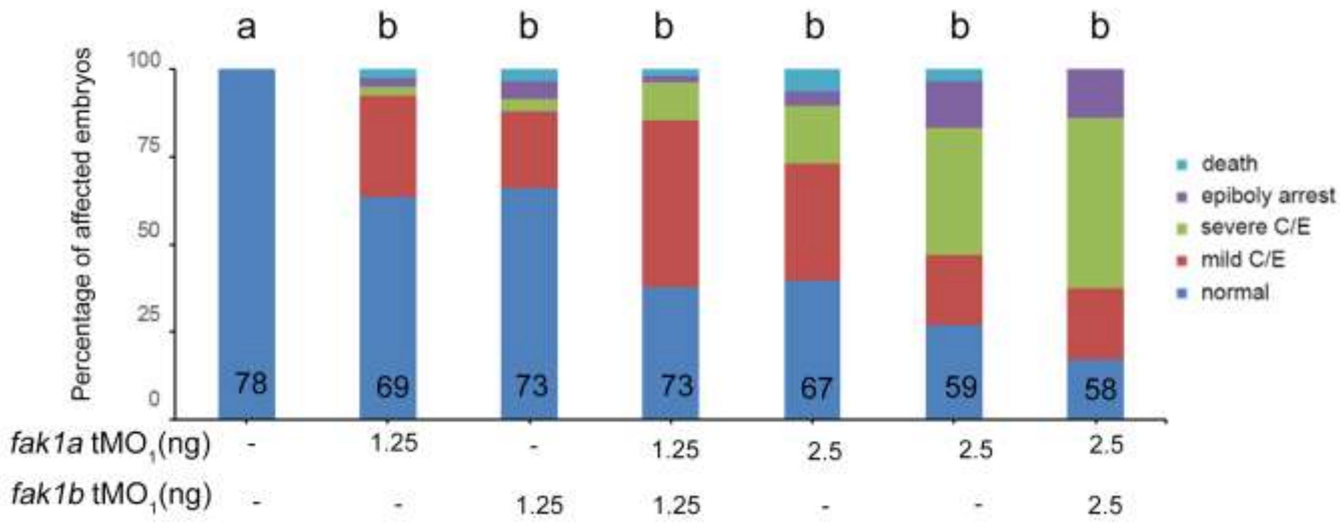


**Fig S11**. *fak1a* and *fak1b* MOs show additive inhibition on gastrulation defect. Embryos injected without or with designated amount *fak1b* $tMO_1$ or *fak1b* $tMO_2$, were cultured, examined, classified and analysed as previously described at bud stage. Quantitative data is shown. The number of embryos analysed are presented in the bottom of each column. Values between groups with a significant difference ($p < 0.05$) are denoted by different letters, N = 3.

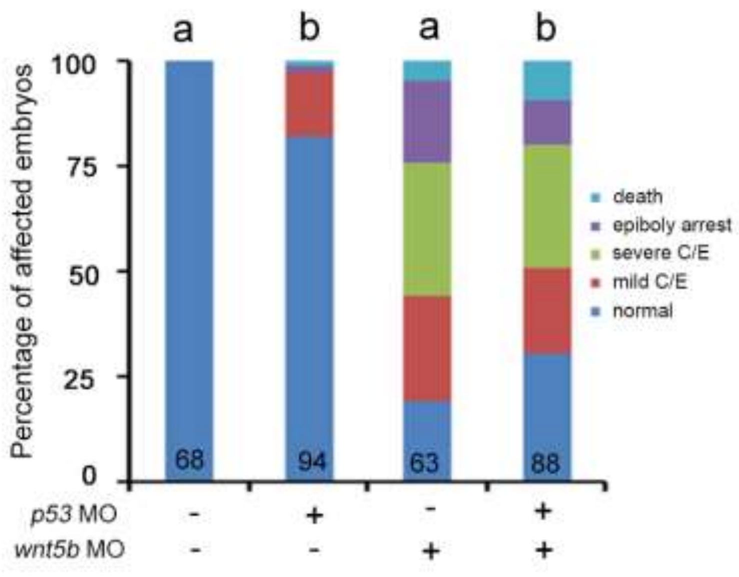


**Figure S12.** Knockdown of *p53* could not rescue epiboly defect in *wnt5b* morphants. Embryos injected with *wnt5b* $tMO_1$ and *p53* MO were examined at bud stage. The percentages embryos with different classes of described gastrulation phenotypes are shown. The number of embryos analyzed is presented at the bottom of each column. Different lettering on the top of each column represents significance difference between groups (N = 3).

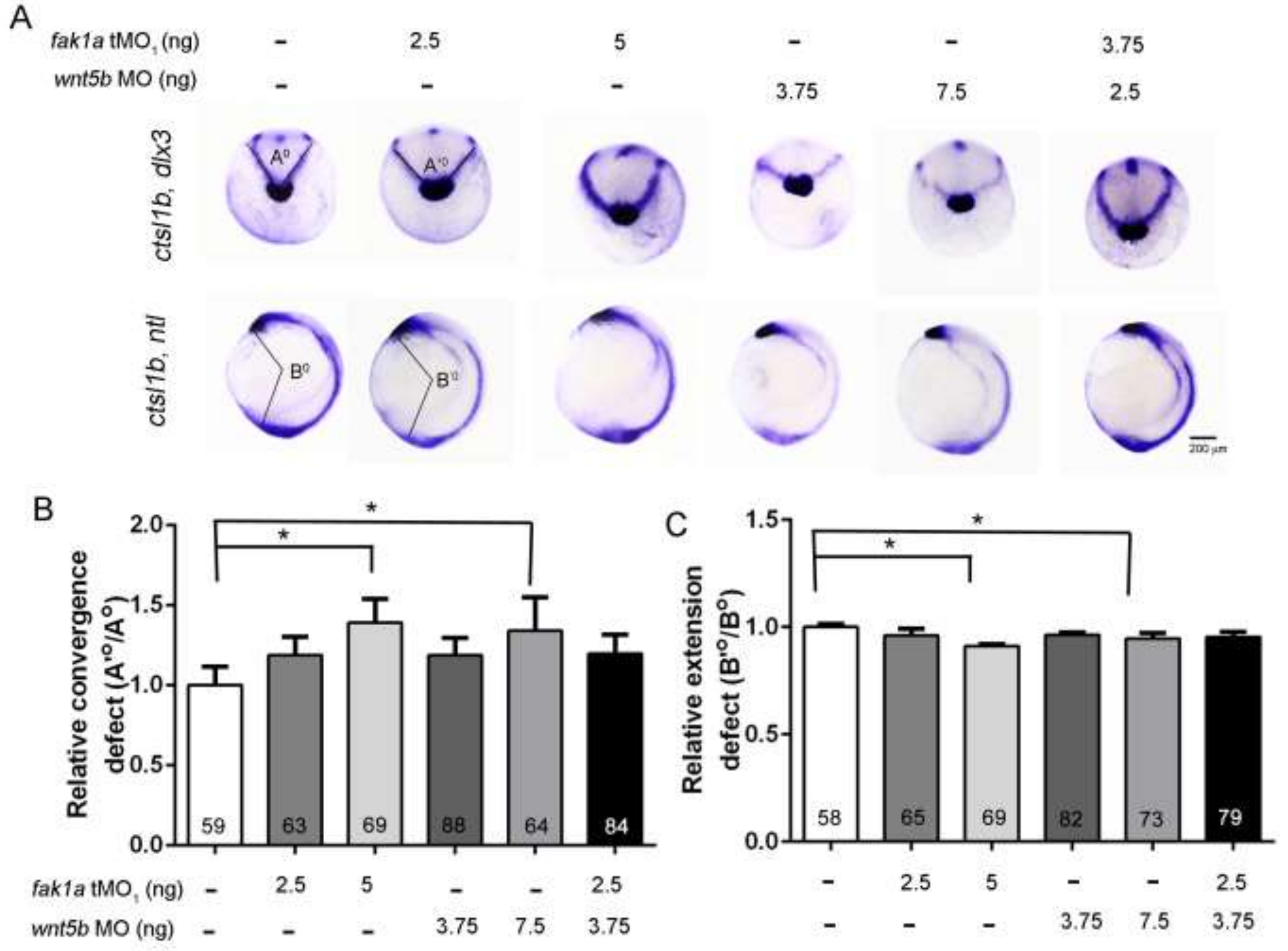


**Figure S13.** *Fak1a* and *wnt5b* showed no synergistic effect during gastrulation. WISH analysis of embryos untreated (control) and injected with different dosages of the *fak1a* translational blocking morpholino ($tMO_1$) and *wnt5b* MO are shown. A: *ctsl1b/dlx3* or *ctsl1b/ntl* staining was respectively used to reveal convergence (A° and A'°, top row) or extension (B° and B'°, bottom row) angles. Lines mark the margin of *dlx3* (black) and *ctsl1b* (black) staining. The relative convergence (B) and extension defects (C) were respectively quantified by calculating the A'°/A° and B'°/B° ratios. $n = 4$, * $p < 0.05$. Scale bar: 200 µm.

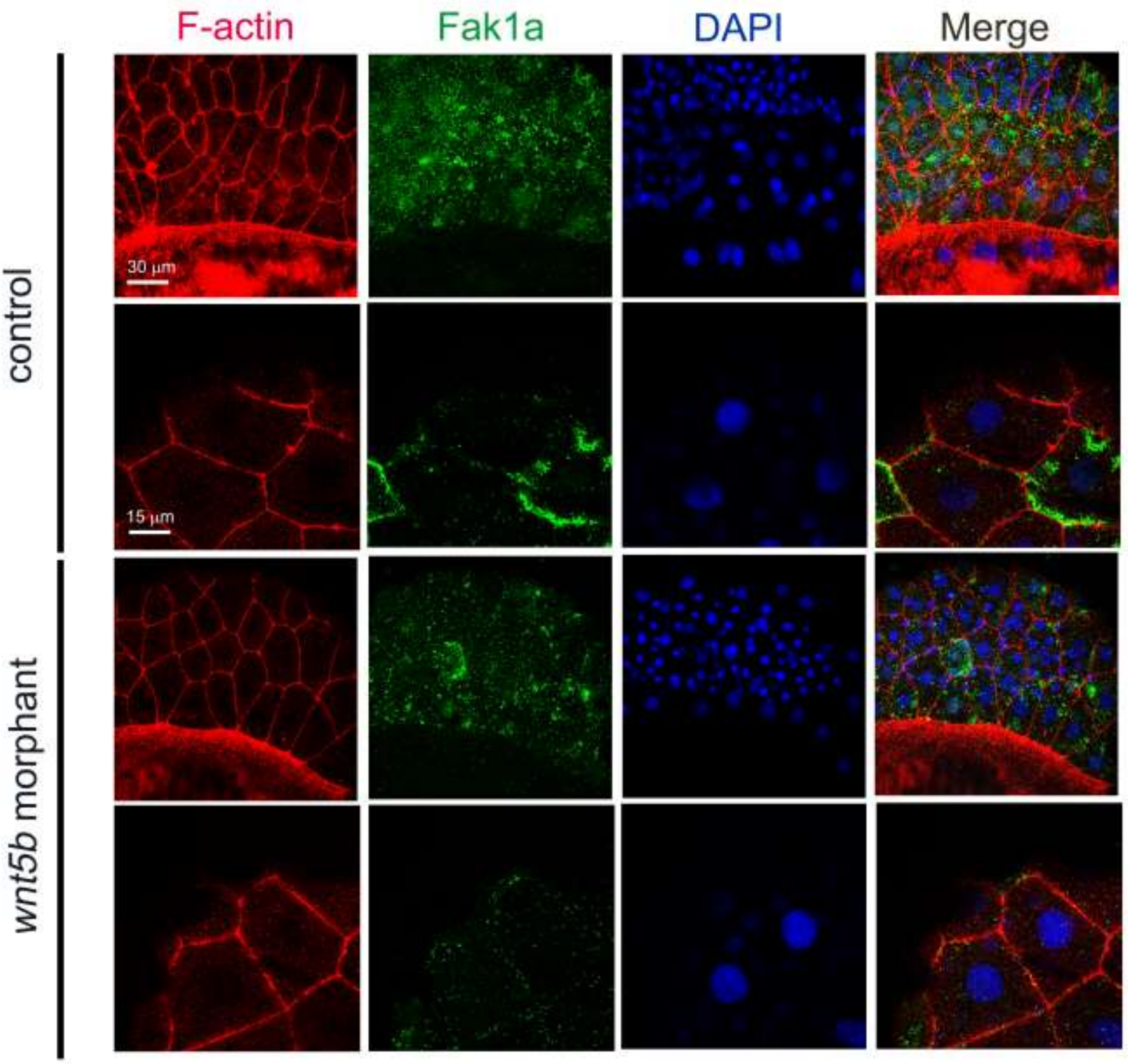


**Figure S14.** Fak1a was downregulated in *wnt5b* morphants. Embryos were untreated or injected with 3.75 ng of the *wnt5b* translational blocking morpholino (tMO), cultured, fixed at the 70% epiboly stage, and then subjected to immunostaining against a Fak1a (C-20) antibody (green), phalloidin (red), and DAPI (blue). Representative photographs and merged images are shown for the whole embryo and region flanking YSL at the indicated magnifications.

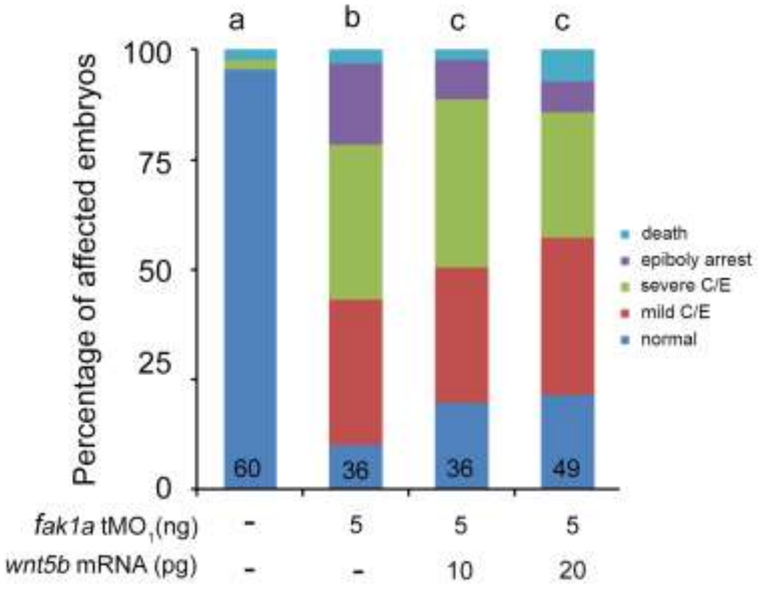


**Figure S15.** *wnt5b* mRNA partially rescued epiboly arrest of *fak1a* morphants. Embryos injected with the *fak1a* translational blocking morpholino ($tMO_1$) or co-injected with *wnt5b* mRNA were observed at the bud stage. Percentages of embryos in different described classifications are shown. Numbers of embryos analyzed are presented at the bottom of each column. Different letters at the top of each column indicate a significance difference between groups ($n$ = 3).

.

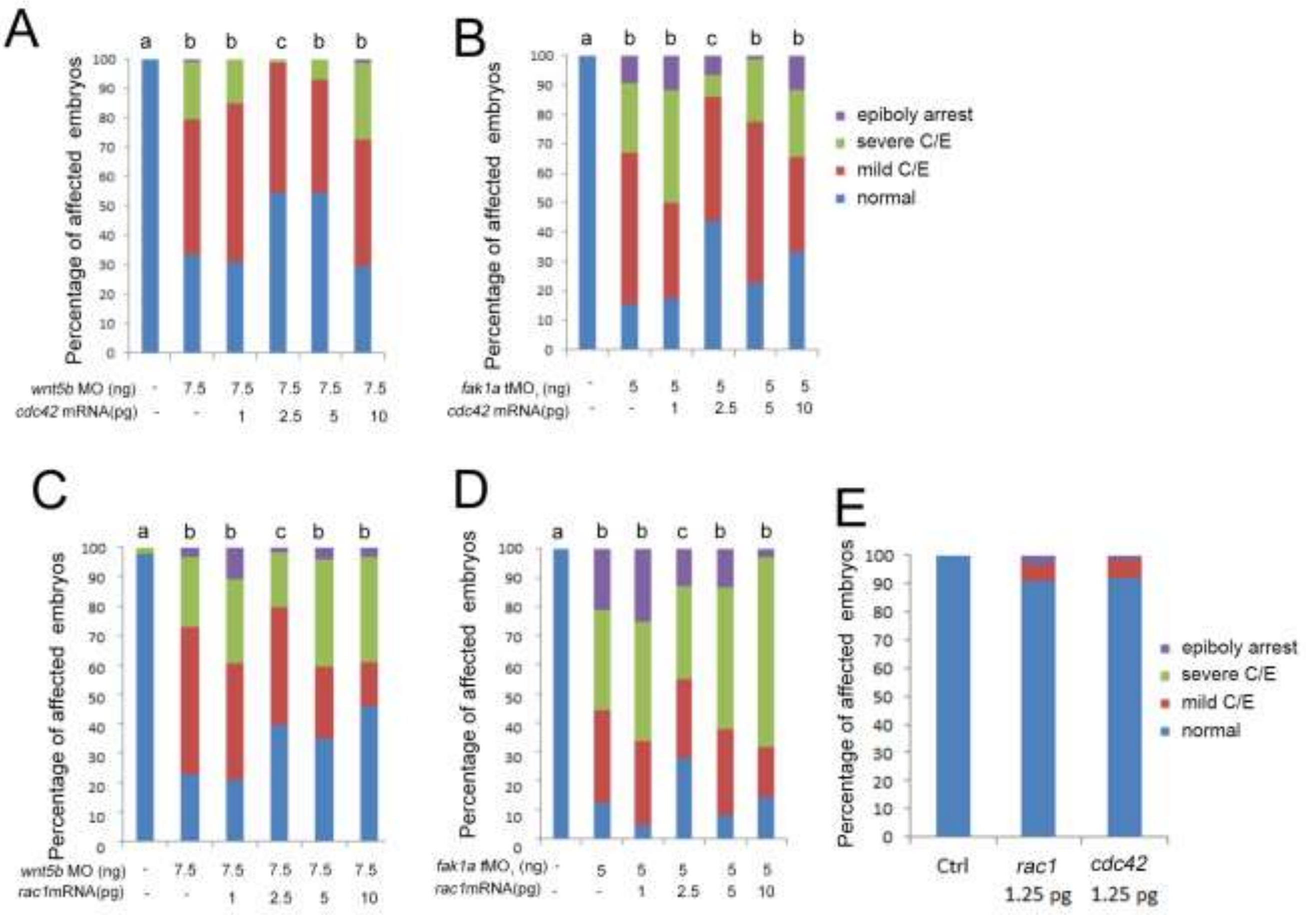


**Figure S16.** Injections of *rac1* and *cdc42* mRNA can rescue gastrulation defects in *wnt5b* and *fak1a* morphants. Embryos were injected with the indicated amount of the *wnt5b* morpholino (MO) (A,C), *fak1a* translational blocking ($tMO_1$) (B,D), *cdc42* mRNA (A,B,E), and *rac1* mRNA (C-E), cultured, and examined at the bud stage to determine the percentage of embryos with previously described gastrulation defects. *n* = 3.

## A: Group images for embryos used in Fig. 9A

| | | |
|---|---|---|
| 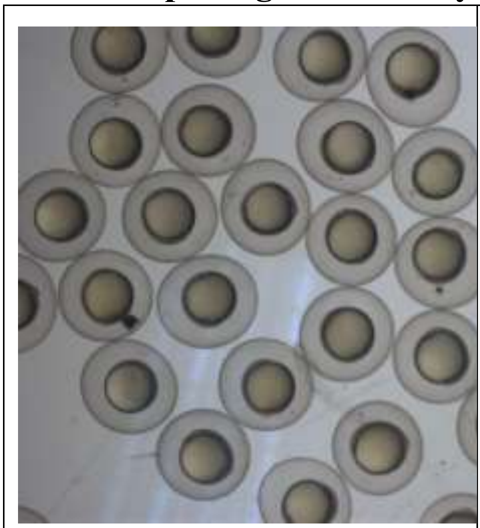 | 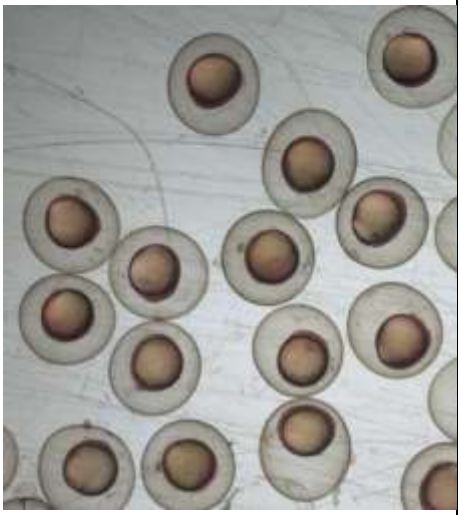 | 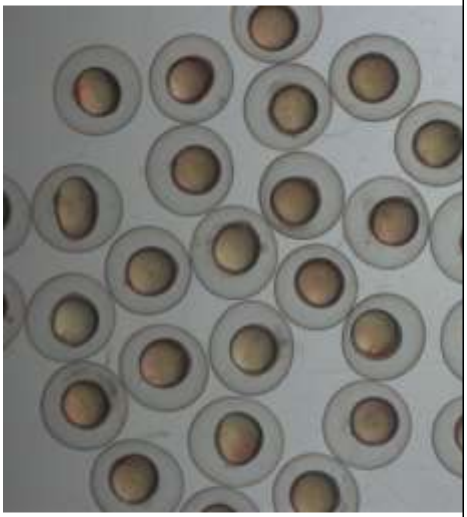 |
| untreated | *wnt5b* MO | *wnt5b* MO + *Rac1* mRNA |
| 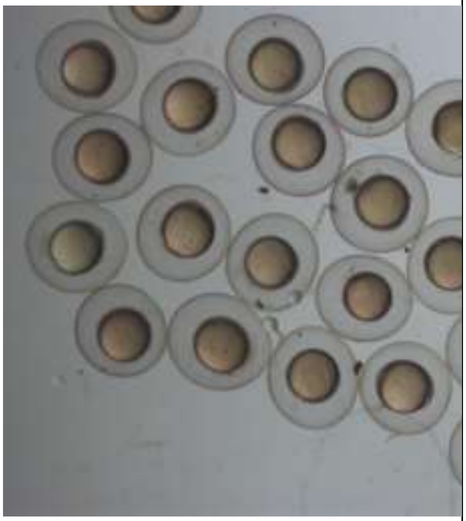 | 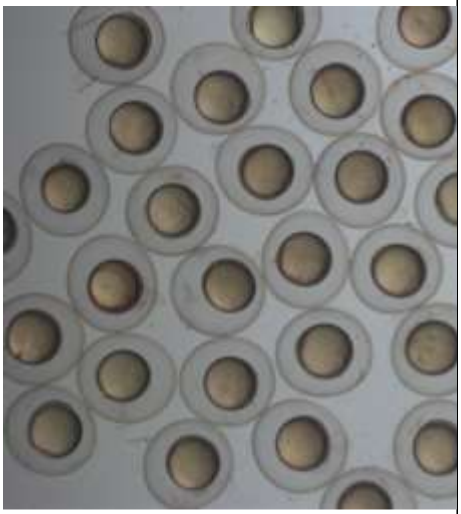 | |
| *wnt5b* MO + *cdc42* mRNA | *wnt5b* MO + *Rac1* mRNA+ *cdc42* mRNA | |

## B: Group images for embryos used in Fig. 9B

| | | |
|---|---|---|
| 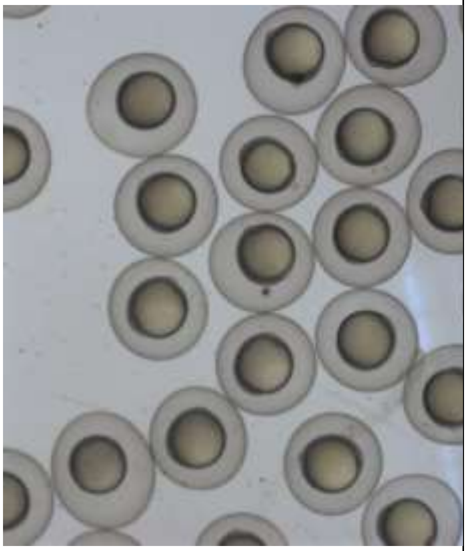 | 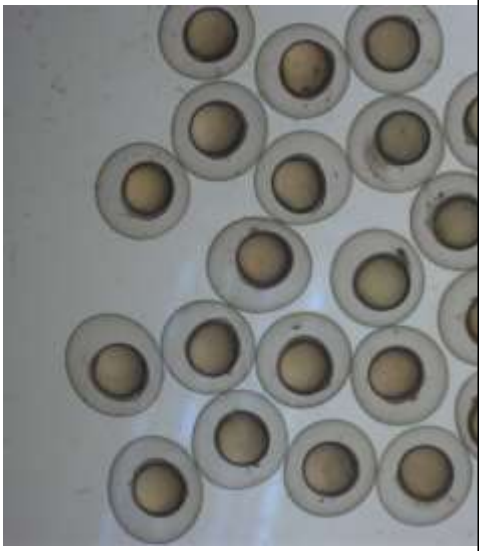 | 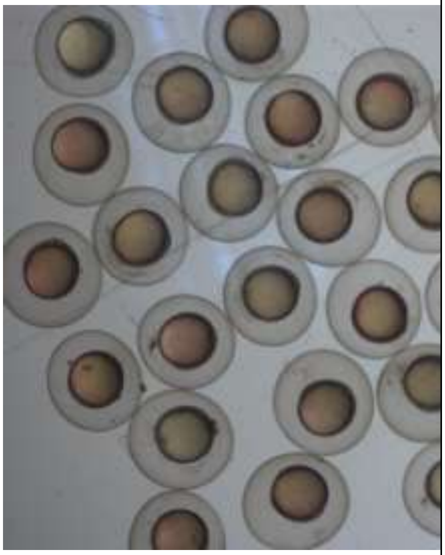 |
| untreated | *fak1a* MO | *fak1a* MO + *Rac1* mRNA |
| 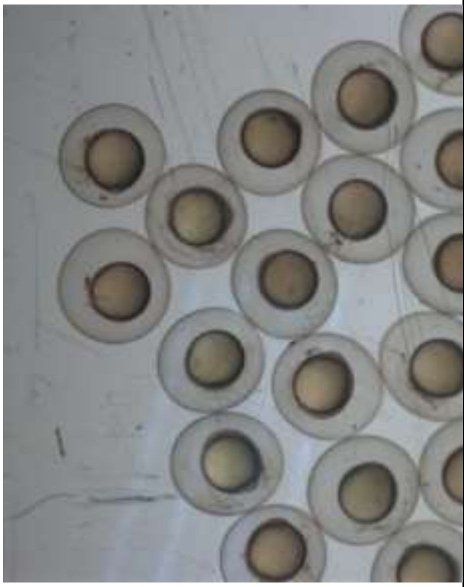 | 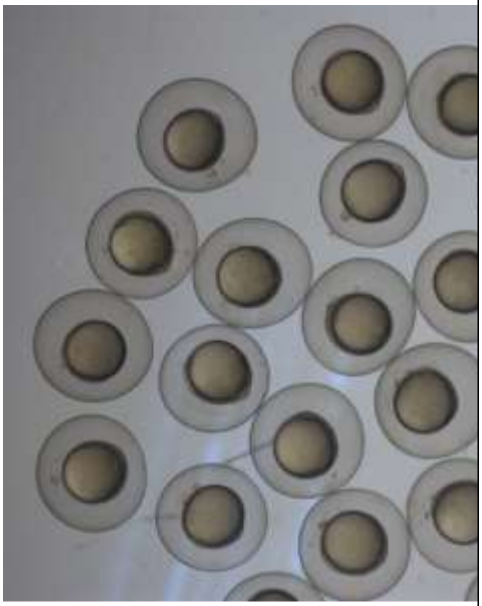 | |
| *fak1a* + *cdc42* mRNA | *fak1a* + *Rac1* mRNA+ *cdc42* mRNA | |

**Figure S17.** Group images embryos used for Fig. 9A (A) and 9B (B).

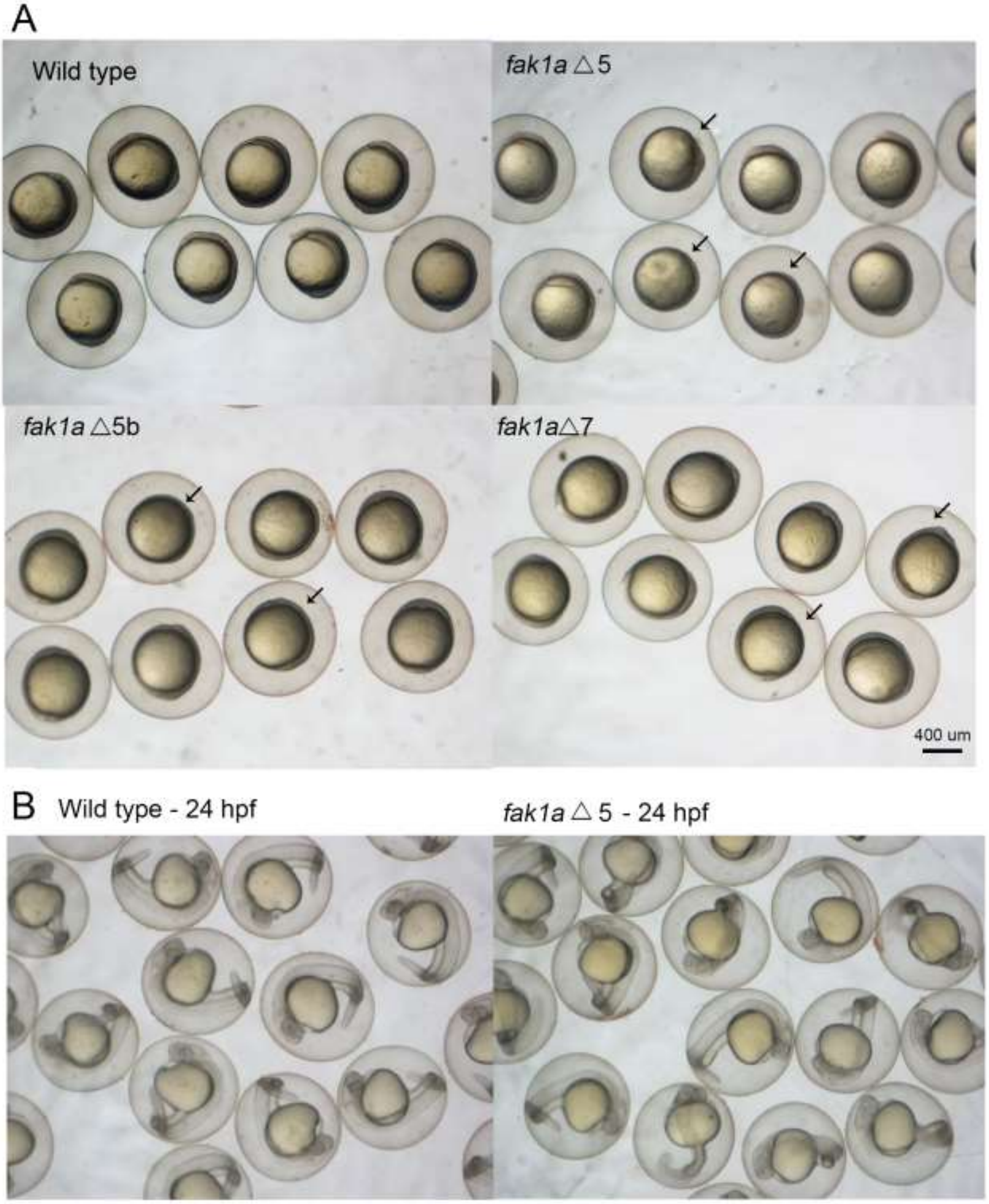


**Figure S18**. Maternal zygotic *fak1a* mutants show mild gastrulation defects. (A) A small proportion (25%) of embryos with three mutant alleles of *fak1a* (Δ5, Δ5b, Δ7) had mild gastrulation defects at the bud stage (indicated by arrows). (B) *fak1a* Δ5 mutants appeared relatively normal at 24 hpf (only Δ5 mutant embryos are shown).

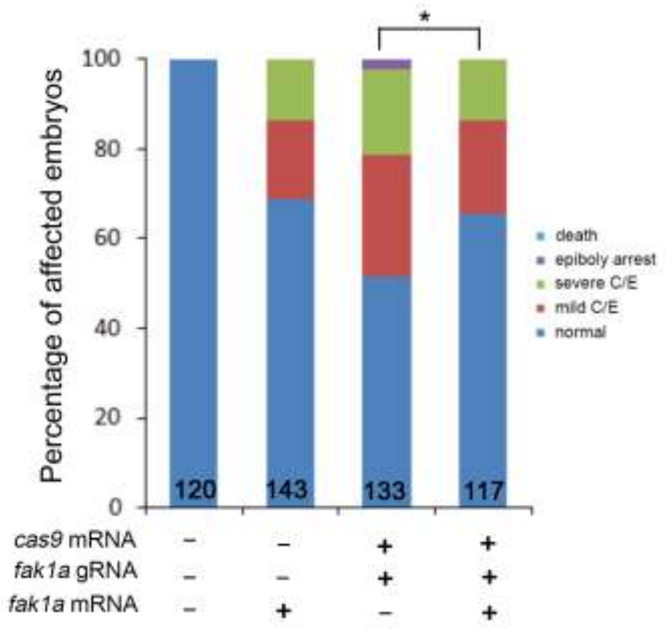


**Figure S19.** Injection of *cas9* mRNA and *fak1a* gRNA causes partial gastrulation defects. Wild-type embryos were injected with 150 pg of gRNA and150 pg of *cas9* mRNA in the absence or presence of 200 pg *fak1a* mRNA at the 1-cell stage and observed in a bright field at the bud stage. Percentages of embryos with different classes of phenotypes as previously described are shown. Numbers of embryos analyzed are given at the bottom of each column. $n = 3$, * $p < 0.05$.

.

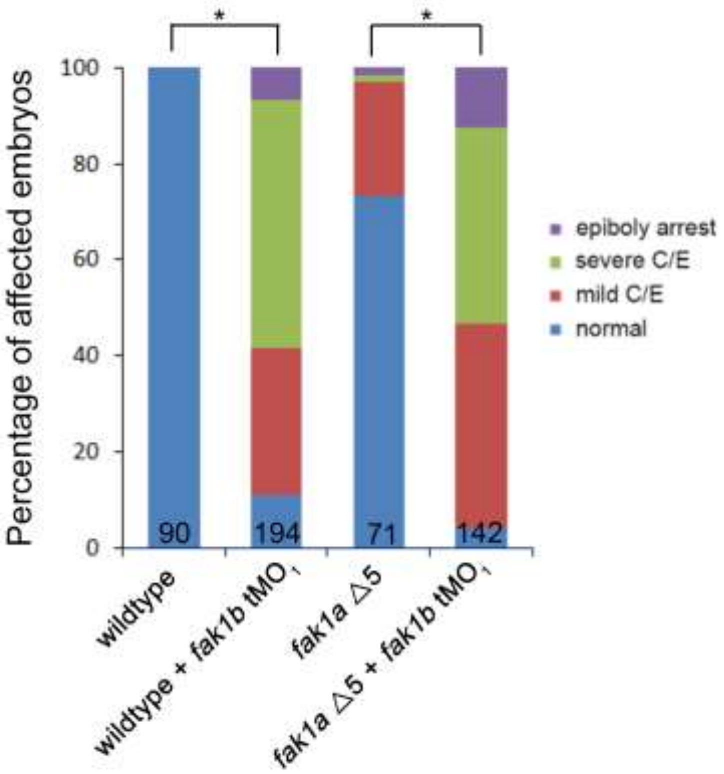


**Figure S20.** *fak1b* morpholino (MO) did not further increase gastrulation defects in *fak1a* mutants. Wild-type (WT) and *fak1a* mutant embryos (Δ5) were untreated or injected with 5 ng of the *fak1b* MO at the 1-cell stage and observed at the bud stage. Percentages of embryos in different classes of the described gastrulation phenotypes are shown. Numbers of embryos analyzed are given at the bottom of each column. $n = 4$, * $p < 0.05$.

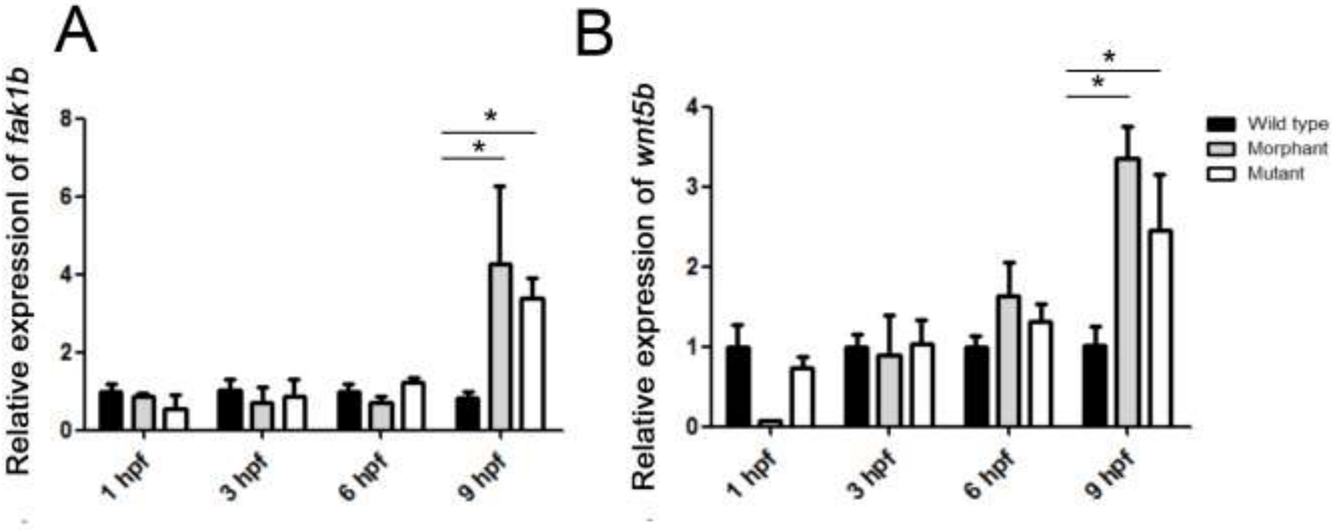


**Figure S21**. Elevation of *fak1b* and *wnt5b* expressions in both *fak1a* morphant and mutant embryos Expressions of *fak1b* (A) and *wnt5b* (B) at designated hours post fertilization (hpf) in wild-type, *fak1a* translational blocking morpholino ($tMO_1$)-injected, and *fak1a* Δ5 mutant embryos were analyzed by a qPCR using *ef1α* as an internal control. Relative expression levels are shown after normalization to their respective expression in wild-type embryos. $n = 4$, * $p < 0.05$.

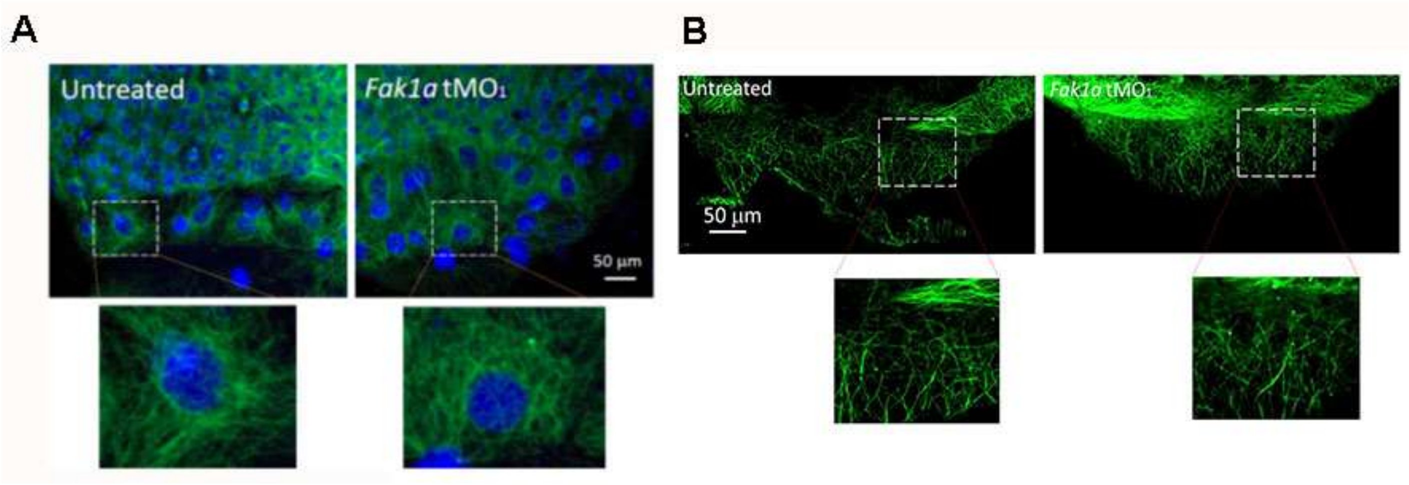


**Fig. S22.** Loss of Fak1b does not affect microtubule structures during epiboly. Embryos were untreated or injected with *fak1a* translational blocking morpholino ($tMO_1$), fixed at 70% epiboly, subjected to immunohistochemistry against alpha-tubulin (1:300 dilution) and stained nuclei by DAPI (N = 3; *n* = 4). We observed no obvious changes in microtubule within perinuclear network (A) and longitudinal arrays within the yolk syncytial layer (B) in *fak1a* $tMO_1$-injected embryos compared to the untreated ones. Enlarged images for the boxed regions are shown below.
